# 2,2′,5,5′-Tetra­methyl-1,1′-(hexane-1,6-di­yl)di-1*H*-pyrrole

**DOI:** 10.1107/S1600536809021965

**Published:** 2009-06-17

**Authors:** Ana C. Santos, Manuela Ramos Silva, Paula V. Monsanto, Ana Matos Beja, Abilio J. F. N. Sobral

**Affiliations:** a Chemistry Department, University of Coimbra, P-3004-516 Coimbra, Portugal; b CEMDRX, Physics Department, University of Coimbra, P-3004-516 Coimbra, Portugal; c Forensic Toxicology Service, National Institute of Legal Medicine, Center Branch, 3000-213 Coimbra, Portugal

## Abstract

The mol­ecule of the title compound, C_18_H_28_N_2_, composed of two 2,5-dimethyl­pyrrole groups linked by a hexane chain, lies across a crystallographic inversion centre. The mean plane of the pyrrole ring is almost perpendicular to the mean plane of the central chain, making a dihedral angle of 89.09 (8)°. The crystal structure is stabilized by inter­molecular C—H⋯π inter­actions.

## Related literature

For the use of chain spacers in conductive polymers, see: Zotti *et al.* (1997 ▶); Chane-Ching *et al.* (1998 ▶); Just *et al.* (1999 ▶). For related structures, see: Ramos Silva *et al.* (2002 ▶, 2005 ▶, 2008 ▶).
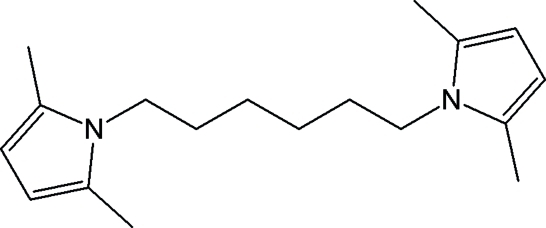


## Experimental

### 

#### Crystal data

C_18_H_28_N_2_
*M* *_r_* = 272.42
Monoclinic, 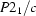

*a* = 7.7608 (3) Å
*b* = 6.4767 (3) Å
*c* = 16.7738 (7) Å
β = 94.309 (3)°
*V* = 840.74 (6) Å^3^
*Z* = 2
Mo *K*α radiation
μ = 0.06 mm^−1^
*T* = 293 K
0.35 × 0.10 × 0.06 mm

#### Data collection

Bruker SMART APEX CCD area-detector diffractometer
Absorption correction: multi-scan (*SADABS*; Sheldrick, 2000 ▶) *T*_min_ = 0.881, *T*_max_ = 0.997
12290 measured reflections
3799 independent reflections
2110 reflections with *I* > 2σ(*I*)
*R*_int_ = 0.025

#### Refinement

*R*[*F* ^2^ > 2σ(*F* ^2^)] = 0.053
*wR*(*F* ^2^) = 0.180
*S* = 1.03
3799 reflections
93 parameters
H-atom parameters constrained
Δρ_max_ = 0.33 e Å^−3^
Δρ_min_ = −0.24 e Å^−3^

### 

Data collection: *SMART* (Bruker, 2003 ▶); cell refinement: *SAINT* (Bruker, 2003 ▶); data reduction: *SAINT*; program(s) used to solve structure: *SHELXS97* (Sheldrick, 2008 ▶); program(s) used to refine structure: *SHELXL97* (Sheldrick, 2008 ▶); molecular graphics: *ORTEPII* (Johnson, 1976 ▶); software used to prepare material for publication: *SHELXL97*.

## Supplementary Material

Crystal structure: contains datablocks global, I. DOI: 10.1107/S1600536809021965/su2118sup1.cif

Structure factors: contains datablocks I. DOI: 10.1107/S1600536809021965/su2118Isup2.hkl

Additional supplementary materials:  crystallographic information; 3D view; checkCIF report

## Figures and Tables

**Table 1:** Hydrogen-bond geometry (Å, °)

*D*—H⋯*A*	*D*—H	H⋯*A*	*D*⋯*A*	*D*—H⋯*A*
C6—H6⋯*Cg*1^i^	0.93	2.67	3.4918 (13)	148

Symmetry code: (i) 
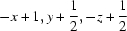
. *Cg*1 is the centroid of the pyrrole ring.

**Fig. 1.**
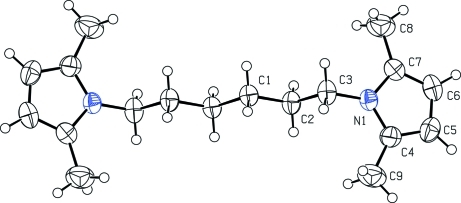
ORTEPII (Johnson, 1976) plot of the title compound. Displacement ellipsoids are drawn at the 50% probabilty level.

**Fig. 2.**
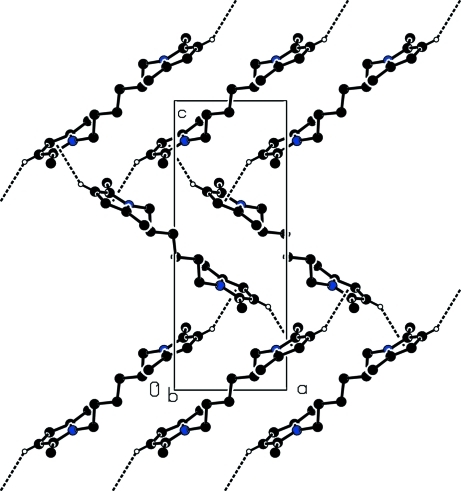
A view down the b axis of the crystal packing of the title compound, showing the C—H···π interactions as dashed lines (see Table 1 for details).

## supplementary crystallographic information

### Comment

Within our project of synthesizing new pyrrole derivatives for several technological purposes (Ramos Silva *et al.*, 2002; Ramos Silva *et al.*, 2005; Ramos Silva *et al.*, 2008), we have prepared the title compound. This pyrrole derivative contains a long alkyl chain between two pyrrole rings. Such a configuration has proven useful in assembling conductive polymer layers (Zotti *et al.*, 1997, Chane-Ching *et al.*, 1998, Just *et al.*, 1999).

The molecular structure of the title compound displays C_i_ symmetry (Fig. 1). The mean plane of the pyrrole ring is almost perpendicular to the mean plane of the central chain; the angle between their mean planes being 89.09 (8)°.

Due to the lack of donors/aceptors there are no conventional hydrogen bonds between the molecules. However, a C—H···π intermolecular interaction, involving the mean plane of the pyrrole ring (Cg1^i^: symmetry operation (i) -x+1, y+1/2, -z+1/2) and hydrogen H6 on atom C6 of the pyrrole ring, links the molecules and they assemble in a herringbone pattern (Fig. 2 and Table 1).

### Experimental

0.250 g (2.15 mmol) of 1,4-phenylenedimethanamine and 0.5 ml (4.25 mmol) of hexane-2,5-dione were dissolved in 20 ml of tetrahydrofuran, under nitrogen atmosphere. 0.086 g (0.339 mmol) of iodine was added to the stirred solution at 40°C. The procedure was monitored by TLC. After completion of the reaction (1.5 h), 20 ml of CH_2_Cl_2_ were added to the mixture. The resulting mixture was washed successively with 5% Na_2_S_2_O_3_ solution (2 ml), NaHCO_3_ solution (2 ml) and brine (2 ml). The organic layer was then dried with anhydrous sodium sulfate and concentrated. The product was purified by flash chromatography on silica gel 60H FLUCKA/dichloromethane and recrystallized in cold dichloromethane, by slow solvent evaporation, yielding needle-shaped crystals; Yield 0.246 grams, corresponding to 0.9 mmol (%) = 21; GC MS (100 µmol/ml in CH_2_Cl_2_) m/*z* = 272; ^1^H-NMR (0.1 *M* in CDCl_3_, 499.428 MHz),σ 1.42 (m, 4H, Methylene), σ 1.62 (m, 4H, Methylene), σ 2.25 (s, 12H, Methyl), σ 3.75 (t, 4H, Methylene, J = 9.99 Hz), σ 5.81 (s, 4H, Pyrrole); ^13^C-NMR (0.1 *M* in CDCl_3_, 125.692 MHz).

### Refinement

H-atoms were positioned geometrically and refined using a riding model: C—H = 0.93 - 0.97 Å with *U*_iso_(H) = k*U*_eq_(parent C-atom), where k = 1.2 for pyrrole and methylene H-atoms, and 1.5 for methyl H-atoms.

### Crystal data

**Table:**

C_18_H_28_N_2_	*F*(000) = 300
*M**_r_* = 272.42	*D*_x_ = 1.076 Mg m^−^^3^
Monoclinic, *P*2_1_/*c*	Mo *K*α radiation, λ = 0.71073 Å
*a* = 7.7608 (3) Å	Cell parameters from 2568 reflections
*b* = 6.4767 (3) Å	θ = 2.6–30.6°
*c* = 16.7738 (7) Å	µ = 0.06 mm^−^^1^
β = 94.309 (3)°	*T* = 293 K
*V* = 840.74 (6) Å^3^	Needle, yellow
*Z* = 2	0.35 × 0.10 × 0.06 mm

### Data collection

**Table:**

Bruker SMART APEX CCD area-detector diffractometer	3799 independent reflections
Radiation source: fine-focus sealed tube	2110 reflections with *I* > 2σ(*I*)
graphite	*R*_int_ = 0.025
φ and ω scans	θ_max_ = 35.4°, θ_min_ = 2.4°
Absorption correction: multi-scan (*SADABS*; Sheldrick, 2000)	*h* = −12→12
*T*_min_ = 0.881, *T*_max_ = 0.997	*k* = −10→10
12290 measured reflections	*l* = −27→26

### Refinement

**Table:**

Refinement on *F*^2^	Primary atom site location: structure-invariant direct methods
Least-squares matrix: full	Secondary atom site location: difference Fourier map
*R*[*F*^2^ > 2σ(*F*^2^)] = 0.053	Hydrogen site location: inferred from neighbouring sites
*wR*(*F*^2^) = 0.180	H-atom parameters constrained
*S* = 1.03	*w* = 1/[σ^2^(*F*_o_^2^) + (0.084*P*)^2^ + 0.0541*P*] where *P* = (*F*_o_^2^ + 2*F*_c_^2^)/3
3799 reflections	(Δ/σ)_max_ < 0.001
93 parameters	Δρ_max_ = 0.33 e Å^−^^3^
0 restraints	Δρ_min_ = −0.24 e Å^−^^3^

### Special details

**Table:**

Geometry. All e.s.d.'s (except the e.s.d. in the dihedral angle between two l.s. planes) are estimated using the full covariance matrix. The cell e.s.d.'s are taken into account individually in the estimation of e.s.d.'s in distances, angles and torsion angles; correlations between e.s.d.'s in cell parameters are only used when they are defined by crystal symmetry. An approximate (isotropic) treatment of cell e.s.d.'s is used for estimating e.s.d.'s involving l.s. planes.
Refinement. Refinement of *F*^2^ against ALL reflections. The weighted *R*-factor *wR* and goodness of fit *S* are based on *F*^2^, conventional *R*-factors *R* are based on *F*, with *F* set to zero for negative *F*^2^. The threshold expression of *F*^2^ > σ(*F*^2^) is used only for calculating *R*-factors(gt) *etc*. and is not relevant to the choice of reflections for refinement. *R*-factors based on *F*^2^ are statistically about twice as large as those based on *F*, and *R*- factors based on ALL data will be even larger.

### Fractional atomic coordinates and isotropic or equivalent isotropic displacement parameters (Å^2^)

**Table:**

	*x*	*y*	*z*	*U*_iso_*/*U*_eq_	
N1	0.36575 (10)	0.90724 (13)	0.13870 (5)	0.0405 (2)	
C2	0.15288 (14)	0.71268 (16)	0.04807 (6)	0.0461 (2)	
H2A	0.2278	0.7239	0.0045	0.055*	
H2B	0.0732	0.8283	0.0444	0.055*	
C1	0.05154 (13)	0.51178 (16)	0.03991 (6)	0.0438 (2)	
H1A	0.1315	0.3970	0.0471	0.053*	
H1B	−0.0269	0.5048	0.0822	0.053*	
C7	0.32211 (13)	1.07945 (16)	0.18158 (6)	0.0444 (2)	
C3	0.26078 (15)	0.72241 (17)	0.12703 (6)	0.0490 (3)	
H3A	0.1842	0.7133	0.1700	0.059*	
H3B	0.3363	0.6029	0.1310	0.059*	
C4	0.53191 (13)	0.92871 (18)	0.11692 (6)	0.0473 (3)	
C6	0.46125 (16)	1.20827 (17)	0.18618 (7)	0.0513 (3)	
H6	0.4681	1.3360	0.2116	0.062*	
C5	0.59257 (15)	1.1140 (2)	0.14579 (7)	0.0547 (3)	
H5	0.7017	1.1684	0.1398	0.066*	
C8	0.15291 (18)	1.1023 (3)	0.21603 (10)	0.0741 (4)	
H8A	0.1485	1.2332	0.2427	0.111*	
H8B	0.1386	0.9932	0.2537	0.111*	
H8C	0.0620	1.0952	0.1740	0.111*	
C9	0.61868 (19)	0.7690 (3)	0.07002 (9)	0.0770 (5)	
H9A	0.7339	0.8135	0.0615	0.116*	
H9B	0.5546	0.7496	0.0194	0.116*	
H9C	0.6235	0.6411	0.0990	0.116*	

### Atomic displacement parameters (Å^2^)

**Table:**

	*U*^11^	*U*^22^	*U*^33^	*U*^12^	*U*^13^	*U*^23^
N1	0.0412 (4)	0.0383 (4)	0.0410 (4)	0.0004 (3)	−0.0041 (3)	−0.0022 (3)
C2	0.0477 (5)	0.0449 (6)	0.0443 (5)	−0.0050 (4)	−0.0057 (4)	0.0003 (4)
C1	0.0452 (5)	0.0434 (5)	0.0420 (5)	−0.0030 (4)	−0.0018 (4)	−0.0029 (4)
C7	0.0486 (5)	0.0423 (5)	0.0413 (5)	0.0083 (4)	−0.0031 (4)	−0.0014 (4)
C3	0.0554 (6)	0.0420 (5)	0.0475 (6)	−0.0075 (4)	−0.0093 (4)	0.0022 (4)
C4	0.0420 (5)	0.0562 (6)	0.0430 (5)	0.0043 (4)	−0.0008 (4)	0.0002 (4)
C6	0.0670 (7)	0.0383 (5)	0.0463 (6)	−0.0021 (5)	−0.0113 (5)	−0.0001 (4)
C5	0.0489 (6)	0.0623 (7)	0.0514 (6)	−0.0127 (5)	−0.0062 (4)	0.0088 (5)
C8	0.0626 (8)	0.0870 (11)	0.0739 (9)	0.0184 (7)	0.0119 (6)	−0.0092 (8)
C9	0.0652 (8)	0.0941 (11)	0.0722 (9)	0.0247 (8)	0.0077 (7)	−0.0169 (8)

### Geometric parameters (Å, °)

**Table:**

N1—C4	1.3735 (13)	C3—H3B	0.9700
N1—C7	1.3830 (13)	C4—C5	1.3647 (17)
N1—C3	1.4529 (13)	C4—C9	1.4904 (17)
C2—C3	1.5141 (14)	C6—C5	1.4049 (18)
C2—C1	1.5212 (14)	C6—H6	0.9300
C2—H2A	0.9700	C5—H5	0.9300
C2—H2B	0.9700	C8—H8A	0.9600
C1—C1^i^	1.5149 (18)	C8—H8B	0.9600
C1—H1A	0.9700	C8—H8C	0.9600
C1—H1B	0.9700	C9—H9A	0.9600
C7—C6	1.3622 (16)	C9—H9B	0.9600
C7—C8	1.4812 (17)	C9—H9C	0.9600
C3—H3A	0.9700		
			
C4—N1—C7	109.16 (9)	H3A—C3—H3B	107.5
C4—N1—C3	125.15 (9)	C5—C4—N1	107.48 (10)
C7—N1—C3	125.29 (9)	C5—C4—C9	129.80 (12)
C3—C2—C1	111.26 (8)	N1—C4—C9	122.72 (11)
C3—C2—H2A	109.4	C7—C6—C5	107.90 (10)
C1—C2—H2A	109.4	C7—C6—H6	126.1
C3—C2—H2B	109.4	C5—C6—H6	126.1
C1—C2—H2B	109.4	C4—C5—C6	108.05 (10)
H2A—C2—H2B	108.0	C4—C5—H5	126.0
C1^i^—C1—C2	113.63 (11)	C6—C5—H5	126.0
C1^i^—C1—H1A	108.8	C7—C8—H8A	109.5
C2—C1—H1A	108.8	C7—C8—H8B	109.5
C1^i^—C1—H1B	108.8	H8A—C8—H8B	109.5
C2—C1—H1B	108.8	C7—C8—H8C	109.5
H1A—C1—H1B	107.7	H8A—C8—H8C	109.5
C6—C7—N1	107.41 (10)	H8B—C8—H8C	109.5
C6—C7—C8	129.72 (11)	C4—C9—H9A	109.5
N1—C7—C8	122.84 (11)	C4—C9—H9B	109.5
N1—C3—C2	114.82 (8)	H9A—C9—H9B	109.5
N1—C3—H3A	108.6	C4—C9—H9C	109.5
C2—C3—H3A	108.6	H9A—C9—H9C	109.5
N1—C3—H3B	108.6	H9B—C9—H9C	109.5
C2—C3—H3B	108.6		
			
C3—C2—C1—C1^i^	−176.89 (11)	C3—N1—C4—C5	−173.23 (9)
C4—N1—C7—C6	0.20 (11)	C7—N1—C4—C9	179.88 (11)
C3—N1—C7—C6	173.26 (9)	C3—N1—C4—C9	6.81 (16)
C4—N1—C7—C8	−178.14 (11)	N1—C7—C6—C5	−0.16 (12)
C3—N1—C7—C8	−5.08 (16)	C8—C7—C6—C5	178.02 (12)
C4—N1—C3—C2	−89.88 (13)	N1—C4—C5—C6	0.06 (12)
C7—N1—C3—C2	98.15 (12)	C9—C4—C5—C6	−179.98 (12)
C1—C2—C3—N1	178.31 (9)	C7—C6—C5—C4	0.06 (13)
C7—N1—C4—C5	−0.16 (12)		

Symmetry codes: (i) −*x*, −*y*+1, −*z*.

### Hydrogen-bond geometry (Å, °)

**Table:**

*D*—H···*A*	*D*—H	H···*A*	*D*···*A*	*D*—H···*A*
C6—H6···Cg1^ii^	0.93	2.67	3.4918 (13)	148

Symmetry codes: (ii) −*x*+1, *y*+1/2, −*z*+1/2.
